# Designing a Multivariate Belt Conveyor Idler Stall Detection and Identification System with Scalability Analysis

**DOI:** 10.3390/s24247989

**Published:** 2024-12-14

**Authors:** Kyeong Su Shin, Younho Nam, Young-Joo Suh

**Affiliations:** 1 Graduate School of Artificial Intelligence, Pohang University of Science and Technology, Pohang 37673, Republic of Korea; ksshin@postech.ac.kr; 2 Department of Computer Science and Engineering, Pohang University of Science and Technology, Pohang 37673, Republic of Korea; younho@postech.ac.kr

**Keywords:** fault diagnosis, anomaly detection, belt conveyor idler, machine learning, wireless sensor networks, scalability

## Abstract

Belt conveyor idlers are freely rotating idlers supporting the belt of a conveyor, and can induce severe frictional damage to the belt as they fail. Therefore, fast and accurate detection of idler faults is crucial for the effective maintenance of belt conveyor systems. In this article, we implement and evaluate the performance of an idler stall detection system based on a multivariate deep learning model using accelerometers and microphone sensor data. Emphasis is place on the scalability of the system, as large belt conveyor installations can span multiple kilometers, potentially requiring hundreds or even thousands of sensor units to monitor. The accuracy of the proposed system are analyzed and reported, along with its network bandwidth and energy requirements. The results suggest that while implementing accurate large-scale idler stall detection is feasible, careful consideration must be paid to observing the available network bandwidth and energy budget in order to avoid prolonged downtimes.

## 1. Introduction

**Figure 1:**
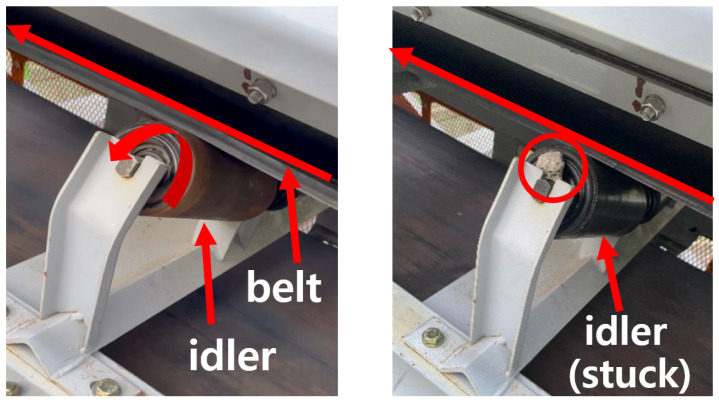
A belt idler moving freely (**left**) and intentionally stalled for depiction and data collection purposes (**right**). The red arrows on the belt and the idler indicate the direction of the movements. Other arrows, circles, and texts are simply used to emphasize important components of the system.

Conveyor belt idlers are cylindrical freely-rotating supports installed along the path of a belt conveyor to support the belt and prevent it from sagging. Idler function is critical to the lifespan of the belt, as the idlers reduce the wear and tear experienced by the belt by supporting it and preventing any drag that could be induced by other structures. Idler failures caused by fault conditions such as failed bearings can cause the idler to stall and drag against the belt. Drag on the belt caused by stalled idlers can induce frictional damage, to the point where the belt or even the whole conveyor system fails (Figure 1).

There have been multiple attempts to implement a system that can automatically detect or predict the idler failures using various sensing technologies. Review articles from Liu and Alharbi [1,2] summarize the current progress of such systems. Earlier designs were often based on the use of thermometers or thermal cameras to identify faults by detecting temperature increases caused by increased friction between the belt and failed idler [3,4]. However, such designs have relatively low sensitivity, and can only detect failure conditions at relatively late stages. More advanced designs often employ vibration sensors, microphones, or acoustic emission sensors along with machine learning (ML) classifiers to detect the vibrational patterns of failed idlers [1,2] (example: [5,6]). Such systems operate by collecting the raw vibration/acoustic sample data from single or multiple sensor units, preprocessing them (usually into spectrograms, wavelet plots, or handcrafted features), then feeding the preprocessed data into ML classifiers. Most works have concentrated on improving the feature extractor and/or model design; for example, a recent manuscript from Alharbi [5] achieved improved detection accuracy by utilizing a pretrained model and an Long Short-Term Memory (LSTM)-based feature extractor.

Unfortunately, due to the challenges and costs associated with installing and maintaining such systems, most real-world belt conveyor operators do not implement such systems, instead simply relying on manual inspections. This is largely due to the scale of belt conveyor installations, with manual inspection usually sufficient for small belt conveyors. For larger installations, such as those seen in mines, steelworks, and power stations, the length of the belt conveyor can span multiple kilometers [7], requiring hundreds or even several thousand sensor units to be installed and maintained. Moreover, these sensor units are often exposed to harsh conditions due to the nature of the tasks involved, and may not have access to high-bandwidth networks or mainline electricity. This makes sensor installation and maintenance challenging tasks, to the point where operators may simply choose not to implement such a system and resort to traditional manual inspection techniques.

This suggests to us that the scalability of these systems needs to be studied and improved in order to allow for wider adaptation of new proposed technologies. Until now, most works have concentrated on improving the accuracy of detection systems, leaving the scalability of these systems somewhat underexplored. The following problems must be solved in order to enable wider adaptation of such technologies: resiliency of the system against sensor failures, network bandwidth requirements, and energy requirements of the system. Because hundreds or thousands of sensor units are exposed to harsh conditions, some sensor failures are expected; thus, the detection system must be able to cope with sensor losses. In addition, if the detection system is based on multivariate sensors, such as accelerometers or acoustic sensors with relatively high sampling rates, then the network bandwidth and energy requirements of the sensor units can grow prohibitively high as the system scales. Thus, the system must be designed and analyzed with the network and energy budgets in mind.

As the size of the system grows, the chance of sensor failure increases. Intuitively, the chance of not experiencing a sensor failure at all at a given moment is $x^{n}$, where *n* is the number of the sensor units and x<1 is the availability of the sensor at that moment (assuming independent and identically distributed probabilities). As the number of sensors grows, this probability decreases rapidly. There have been several studies on diagnosing sensor faults and mitigating the effects of failures [8,9,10]. These articles mainly concentrate on topics such as detecting sensor failures themselves [8], identifying typical failure modes, and implementing rule-based or machine learning-based sensor failure detection models, while some other articles concentrate on replacing faulty sensor data samples with synthetic values (data imputation), which assumes that the sensor failure has been already identified [9]. For example, in case of a multivariate system, a simple nearest-neighbor estimator can be used to deduce the value of a missing sample by using data samples from other operational sensor units [9].

Another possible problem involves the network bandwidth and the energy requirements. Having multiple sensors over a large area means that it is often impractical to tether all of them together into the power line and a wired network or shared data acquisition (DAQ) unit. This means that the sensors may have to be battery-powered and transport data samples using wireless technologies, which can imply severe network bandwidth and energy restrictions. This is not necessarily a problem when the sensor units operate at a relatively low sampling rate; some temperature-based idler fault detection systems can indeed operate purely with harvested energy [11]. However, such systems have relatively low sensitivity, and high-sensitivity detectors based on vibration/acoustic emission sensors and ML classifiers are generally much more energy intensive. Furthermore, for joint classification, measurements from multiple sensor units need to be transmitted into a central data collection unit prior to classification. As deep learning-based sensing systems may need access to the raw unprocessed (or minimally processed) streams of sensor readings for feature extraction [12], the bandwidth and the energy requirements associated with data transmission can grow to an impractical level, as each sensor unit could generate and transmit up to a few megabits of data samples per second (as demonstrated in later sections).

Therefore, naively transmitting every collected data sample can quickly become impractical in the case of deep learning-based multivariate sensing systems, and data reduction techniques may be needed in order to keep the network bandwidth and energy budgets at a manageable level. Some systems use duty-cycled sensor data to reduce the energy requirements [13,14]; in these articles, the authors try to balance the system accuracy and energy efficiency of the system by carefully adjusting the duty cycle (on–off periods) of the involved sensor units. Others have used compressive sensing techniques such as sub-Nyquist sampling [15,16] or edge computing techniques such as autoencoders to compress the data [17]. These approaches try to reduce the amount of data generated by the sensor units while minimizing the loss of information by either adjusting the sampling rates or by applying lossy compression to the data. Another approach to the problem is to not install sensor units directly onto the belt conveyor, but to install them on robots or unmanned vehicles which scan the belt conveyor physically in prescheduled intervals [11]. However, such systems come with challenges of their own; for instance, because the sensor units are not directly attached to the belt, and the vehicles themselves generate vibrational and acoustic noises, the quality of the data can be degraded.

In this article, we implement and evaluate a belt conveyor idler monitoring system that detects and identifies stalled idlers by using accelerometers and microphones as cheaper substitutes for piezoelectric transducers. We analyze the reliability, network bandwidth, and energy requirements of the proposed system to evaluate the practicality of the design. Wired accelerometers and microphones installed on our belt conveyor testbed are used to collect near-ideal-condition datasets. The data samples are then intentionally modified to simulate sensor failures and measure the effect of the accelerometer/microphone sampling rates on the accuracy of the model. The results of our analysis suggest that such systems can indeed detect and locate stalled idlers accurately; however, careful consideration should be given to network and energy efficiency so as not to exceed the network and energy budgets.

The main contribution point of this article is the scalability analysis results, which address how well a deep learning-based idler fault detection model can scale in terms of network bandwidth and energy costs as well as the system’s survivability when exposed to harsh operating conditions under which some sensor units can suffer failures.

The rest of this paper is organized as follows: in Section 2, we discuss the methods and equipment used to collect the dataset and the models used to implement the idler stall detection and identification system; in Section 3, we display the experimental results of the proposed system, including the model accuracy, network bandwidth, and estimated energy requirements; in Section 4, we analyze and discuss the obtained results to derive conclusions; finally, we summarize our results and wrap up the article in Section 5.

## 2. Materials and Methods

### 2.1. Data Collection

**Figure 2:**
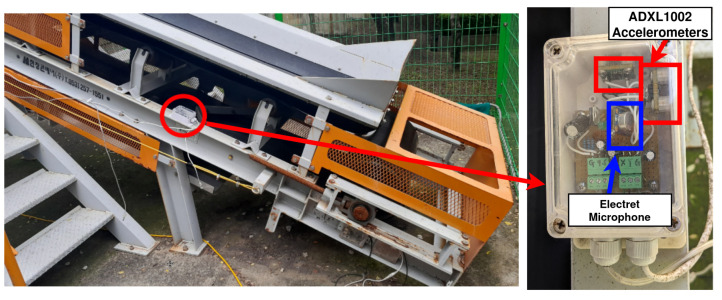
Design of the sensor units and their installed locations.

We collected datasets from our own belt conveyor testbed, consisting of a sloped v-trough belt conveyor with an upper belt length of 7 m. Analog accelerometers and microphones were installed on the frame of the belt conveyor to collect data samples, as shown in Figure 2. To simulate stalled idlers, the idlers were intentionally jammed with stones (Figure 1). When jammed in this way, the idlers were unable to rotate freely and dragged against the belt.

#### 2.1.1. Hardware Setup

Vibration and acoustic data samples were collected using ADXL 1002 Micro-Electro-Mechanical Systems (MEMS) analog single-axis accelerometers (from Analog Devices, Wilmington, MA, USA), and electret microphones with Maxim Integrated MAX4466 amplifier modules (by Adafruit, New York, NY, USA) by connecting them to a NI-6353 DAQ unit, from National Instruments (Austin, TX, USA) with generic shielded cables. MEMS accelerometers and electret microphones were used as substitutes for piezoelectric accelerometers and acoustic emissions sensors, which are often preferred in academic research projects; the rationale behind this choice was that these types of sensors are more likely to be used in real-world deployments. While piezoelectric transducers usually provide higher sensitivity and larger bandwidth than their MEMS counterparts, they also come at much higher cost, which is difficult to justify for large-scale deployments involving hundreds to thousands of sensor units. As shown in the next section, the data quality of these MEMS accelerometers and electret microphones is mostly sufficient for our purposes. All equipments and sensor modules were imported to Pohang, South Korea via local electrical component suppliers.

Two accelerometers and one microphone were grouped to form a ‘sensor unit’. Each ‘sensor unit’ was then enclosed in a weatherproof enclosure for outdoor installations, as shown in Figure 2. The accelerometers were pointed perpendicular to the frame, one pointing upward and the other pointing to the left, forming two orthogonal measurement axes. Because the microphone is an omnidirectional unit, it receives sound waves from every direction. In total, ten sensor units were produced and installed, providing thirty analog measurement channels in total (twenty accelerometers and ten microphones). The sensor outputs were directly sampled by a 32-channel 16-bit DAQ NI-6353 at a sampling rate of 31.25 kSa/s, which is the maximum sampling rate achievable by the DAQ when all 32 channels are scanned simultaneously. This is reasonably close to the Nyquist rate of the sensors [18,19], which is 22 kSa/s and 40 kSa/s for the accelerometers and microphones, respectively. All 32 channels of the DAQ were scanned, with 30 channels actually connected to the sensors and the two remaining unused channels connected to dummy resistors to suppress ghosting effects caused by the voltage range differences between the microphones and accelerometers [20]. The collected samples were stored in files using SigMF format version 1.0 [21], then transmitted to a centralized data server for further analysis. Python 3.11.2 and the nidaqmx Python library (version 0.9.0) were used to implement the collection software.

**Figure 3:**
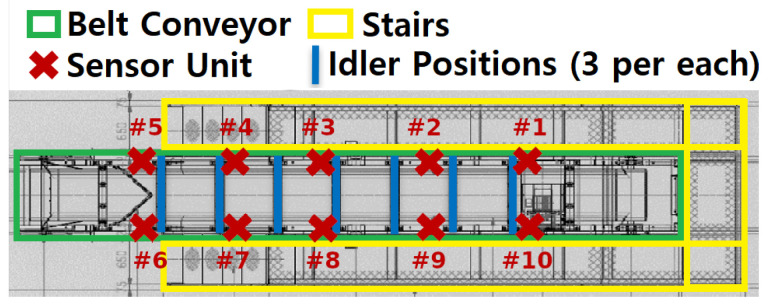
Locations of the sensor units and idlers. Each sensor unit contains three sensors (two accelerometers and one microphone), while each idler location contains three idlers (left, middle, and right idler). The numbers (#1–#10) denote the identification numbers of the sensor units used internally.

The locations of the sensor units are shown in Figure 3. The separation between the sensor units along the frame were approximately 1.2 m, with some variations in placement due to various physical restrictions.

**Figure 4:**
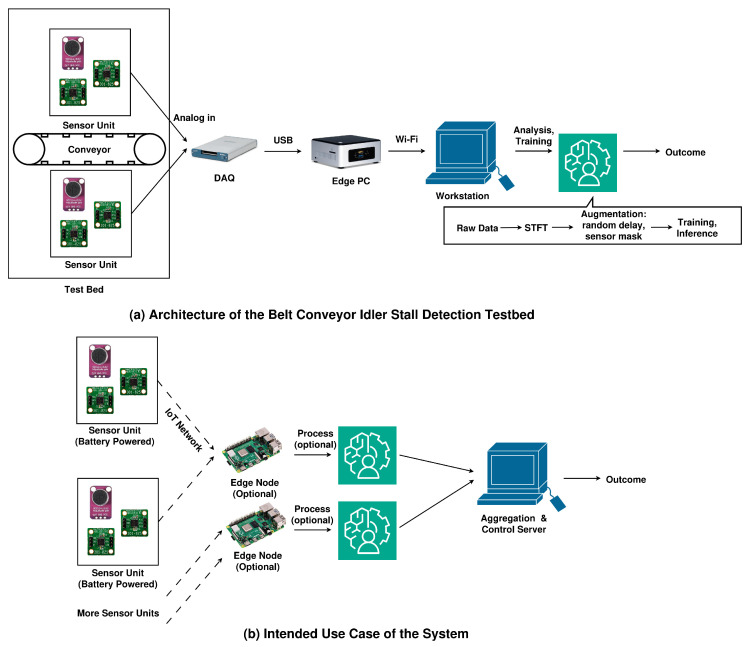
Overview of the system: (**a**) system architecture of the testbed used to collect the data samples and (**b**) expected system architecture in actual deployment.

The overall system architectures are shown in Figure 4. It must be noted that the testbed that we used to collect the datasets and train and evaluate the models was structured differently from the expected deployment scenarios, especially in that the testbed relies on wired sensor units instead of wireless units. This was done intentionally in order to collect high-quality samples without duty cycling. The data samples were intentionally unsynchronized later on during the training and evaluation phases to simulate unsynchronized wireless sensor units.

#### 2.1.2. Dataset Collection

Two types of data samples were collected: normal samples, which formed the normal dataset, and samples with a stalled idler, which formed the anomaly dataset. The normal dataset contained signal samples collected during the normal operation of the belt conveyor with no stalled idlers, while the anomaly dataset contained signal samples with one stalled idler at a known position. As shown in Figure 1, the idler was intentionally jammed to cause a stall. Due to physical constraints, only the upper left/right idlers were jammed (seven left idlers and seven right idlers, for a total of fourteen). The belt was not loaded with payloads during data collection.

The list of collected datasets is summarized in Table 1. The speed of the belt is directly related to the inverter frequency, with the slowest at 10 Hz (approximately 0.11 m/s) and the fastest at 60 Hz (approximately 0.63 m/s).

### 2.2. Model Implementation

#### 2.2.1. Design Considerations

State-of-the-art idler fault detection systems usually rely on ML algorithms to process sensor data and to identify faults [2]. While the properties of one of the most common failure modes, namely, bearing faults, have been closely analyzed and can be inspected algorithmically [22], this is not the only possible failure mode, and the observed power spectra can be vastly different even in the case of bearing faults. This is because the vibrations and acoustic noises created by the belt conveyor are not solely from the bearings, but from various interactions; for example, the drag induced by the stalled bearing can also generate various frequency components in the accelerometer and microphone sensor data which cannot be easily accounted for.

Various neural network-based ML models have been considered, including simple multilayer perceptrons, 1D convolutional neural networks (CNN), recurrent neural networks, and 2D-CNNs. In addition, various preprocessing techniques for the samples have been considered, including the discrete Fourier transform (DFT) for ML models requiring 1D data shapes and the short-time Fourier transform (STFT) and continuous wavelet transform for ML models requiring image-like 2D input data shapes. We selected a 2D-CNN, as it showed the most promising results during our preliminary analysis.

It must be noted that STFT-based models tend to require more sample points to make an inference than the other alternatives considered above. This is because the number of sample points needed to calculate an STFT matrix is equal to twice the number of the elements in the matrix when no overlaps are assumed. Wavelet transform-based models and multivariate 1D time series-based models tend to require fewer sample points to make an inference, although this is implementation-dependent and not necessarily always the case. Nonetheless, this means that our STFT-based model is likely to suffer from higher network overhead than alternative designs. While this is not favorable from the viewpoint of scalability, proving the practicality of the model with STFT would also demonstrate the practicality of the system with smaller models that would require less network bandwidth to operate.

#### 2.2.2. Model Design

Multivariate time series sensor data streams are converted into time-frequency matrices with STFT, then inputted to a 2D-CNN. MobileNet V2 [23] was modified to allow 30-channel STFT input data for use as our classification model. This model detects whether an idler stall has occurred; in the event of a fault, it also identifies the location of the stalled idler. The output of the model (the expected label) is either 0 if no idler is stalled, or the position of the stalled idler (1–14) if a stall is detected. Python 3.11.2 and PyTorch 2.0.1+cu117 were used to implement the model.

#### 2.2.3. Data Augmentation for Sensor Fault Tolerance

Augmented samples are used to train the model. The channels of the input data were randomly selected and masked with zeros to simulate sensors that are missing due to sensor malfunctions or communication failures caused by network instability. A discrete uniform distribution with a probability of 0.5 was used to select and drop the channels (sensors). In this way, the trained model learns to deal with missing sensor data.

We assumed that failed sensor units transmitted either no data or data consisting of only zeros. While this is not necessarily true, similar effects can be achieved by implementing a sensor failure detection system [8] and filtering out any faulty samples detected with it.

Every random parameter used in the augmentation process was recalculated every time the augmentation was applied in order to maximize the randomness of the results.

#### 2.2.4. Preprocessing

The augmented samples were converted into time–frequency domain matrices by applying STFT. The elements in the STFT matrices were then squared to obtain power estimates, converted to the logarithmic scale (base-10), and normalized to approximately [0, 1] using pre-estimated normalization factors. The resulting matrices were used as the input features of the CNN model.

#### 2.2.5. Training and Evaluation

The implemented model was trained using the collected data samples split in an 8:1:1 training–validation–testing ratio. Supervised learning was used to train the model. The hyperparameters and various configurations used to train the model are displayed in Table 2.

Samples with an inverter frequency of 30 Hz were intentionally excluded in order to evaluate the generalization performance of the model. All other samples were used as either training samples, validation samples, or testing samples. The excluded 30 Hz samples were used later to test the accuracy of the model on unseen environments (unseen inverter configurations), and are referred to as the “unseen test data”.

In addition to the main identification model, an additional model was trained with the augmentation step disabled in order to evaluate the effectiveness of the augmentation in hardening the model. In addition, several more models were trained with decimated samples to evaluate the minimum acceptable sampling rates of the system. Two different downsampler configurations were considered: the first with an ideal (DFT-based) low-pass filter, and the second without the low-pass filter, potentially allowing high-frequency components to be aliased into low-frequency regions.

## 3. Results

### 3.1. Network Bandwidth and Energy Requirements

The network and energy usages of the sensor units were calculated to evaluate the scalability of the system.

#### 3.1.1. Theoretical Limits

The theoretical bound of the network bandwidth and energy requirements (of the communication circuits) can be estimated using the Shannon capacity and a channel model.

The Shannon capacity of a wireless channel is

(1) $$C=B\log_{2}\left(1+\frac{S}{N}\right),$$

where *C* is the channel capacity (bits/s), *B* is the bandwidth of the channel (in Hz), and *S*/*N* is the signal-to-noise ratio of the received signal. The available bandwidth is usually controlled by government agencies (the FCC in the case of the U.S.), and is not under our control. The noise power *N* is higher than or equal to the Johnson–Nyquist noise, which is approximately −174 dBm/Hz (linear-log scale conversion may be needed). Finally, the signal power *S* is approximated by dividing the power loss by the transmission power (assuming a linear scale), which is environment-dependent. If free (open) space is assumed, then the free space path loss equation can be used to estimate the loss:

(2) $$\frac{P_{r}}{P_{t}}=D_{t}D_{r}{\left(\frac{\lambda }{4\pi d}\right)}^{2}$$

where $P_{r}$ is the received power (linear scale), $P_{t}$ is the transmitted power, $D_{t}$ is the transmitter antenna gain, $D_{r}$ is the receiver antenna gain, λ is the signal wavelength, and *d* is the distance between the transmission and reception antennas.

By plugging this in to the Shannon capacity and integrating the capacity equation, it is possible to estimate the minimum energy required to transmit the data.

However, the estimated results can be significantly off from the real-world results, as the exact path loss model and various energy overheads experienced by the transceivers are not known. Thus, instead of using the theoretical bounds, we simply use empirical efficiency figures advertised by Wi-Fi HaLow [24] in the rest of this article. Wi-Fi HaLow is a variation of the Wi-Fi standard optimized for long-distance machine-to-machine communication systems. The claimed energy efficiency of a typical Wi-Fi HaLow node is 22.4 kbits/J [24], that is, 22.4 kbits of data transmission per 1 joule of energy.

The physical layer of Wi-Fi HaLow is based on a modified IEEE 802.11ac [25] physical layer (PHY), downclocked to 10% of the original speed of the IEEE 802.11ac; therefore, the theoretical maximum throughput of a Wi-Fi HaLow PHY is approximately 86.7 Mbps, which is one-tenth the maximum throughput of IEEE 802.11ac. The realistic maximum throughput is usually much lower, probably somewhere around 10 Mbps depending on various environmental factors.

#### 3.1.2. Continuous Data Streaming

The minimum network bandwidth required by our system to stream every sample into the centralized data collector server continuously without downsampling or duty-cycling is as follows:

(3) 31,250Sa/s×16bits/Sa×30channels=15mbps.

The required network bandwidth per sensing channel to transmit every sample would then be

(4) 31,250Sa/s×16bits/Sa=0.5mbps.

In our case, the system managed to stream every sample to our centralized server over Wi-Fi in real time without issue; however, such luxuries cannot be expected in more realistic large-scale belt conveyor installations. For example, as discussed above, the 15 Mbps bandwidth requirement is fairly close to the achievable limit of a realistic Wi-Fi HaLow network. A typical 5G New Radio (NR) Enhanced Mobile Broadband backhaul can support approximately 100 Mbps of uplink speed [26], which is lower than the downlink speed due to the asymmetricity of the uplink and downlink connections. This means that a high-performance 5G modem should be able to handle approximately 200 sensor channels (accelerometers or microphones) in our setups without applying data reduction techniques. Adding additional transceivers may not necessarily improve the situation, as the spatial diversity can quickly become the limiting factor. In such cases, data reduction (duty cycling, downsampling, compression, etc.) is unavoidable. Other IoT-optimized wireless communication protocols such as ZigBee and LoRa have very limited uplink bandwidth and do not meet our bandwidth requirements, as these protocols are generally not designed to carry raw data samples directly.

#### 3.1.3. Per-Inference Network Requirements

To create the 256 × 256 × 30 spectrogram used by our inference model, we need to transmit

(5) 256×256×30×2×2bytes/Sa=7.5MiB

of raw data to the central processing server. Similarly, the per-channel cost of the system is 0.25 MiB/ch. Using the 22.4 kbits/joule energy efficiency figure from Wi-Fi HaLow [24], the required amount of energy can be estimated as follows:

(6) $$\frac{7.5\;\mathrm{MiB}}{22.4\;\mathrm{kbits}/J}=2740\;J\;=\;0.76\;\mathrm{Wh},$$

amounting to approximately 91.4 J (0.025 Wh) per sensing channel, or 0.076 Wh for each sensor unit with three sensing channels.

### 3.2. Data Visualization

**Figure 5:**
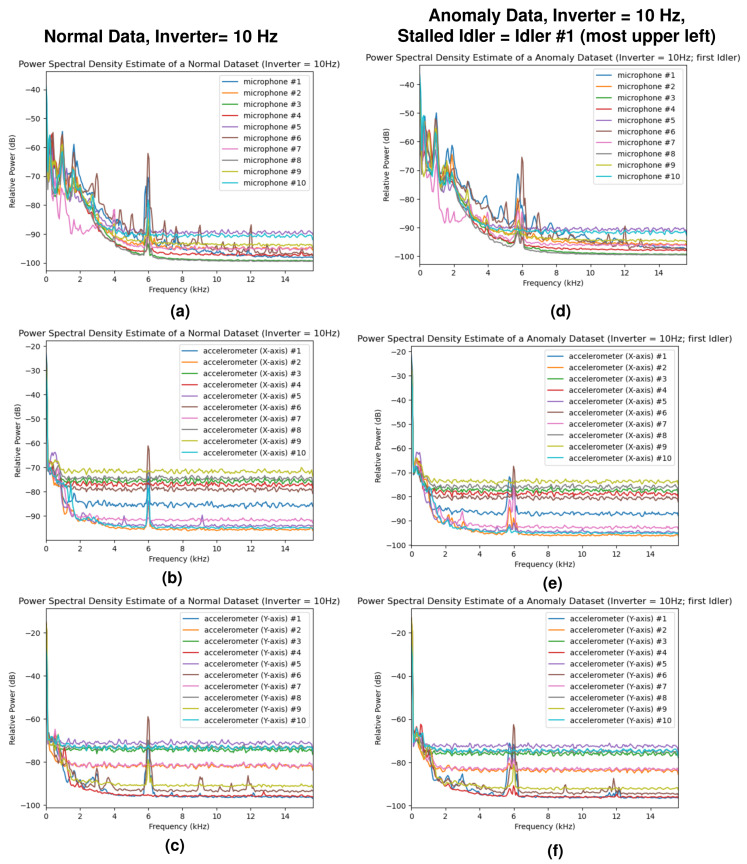
Power spectral density (PSD) estimates of the sensor data for an inverter frequency of 10 Hz: (**a**,**d**) PSD estimates of the microphone sensors for the normal and anomaly datasets, (**b**,**e**) PSD estimates of the *x*-axis accelerometer sensors, and (**c**,**f**) PSD estimates of the *y*-axis accelerometer sensors. The sensor numbers (#1–#10) in the plots above correspond to the sensor identification numbers in Figure 3.

**Figure 6:**
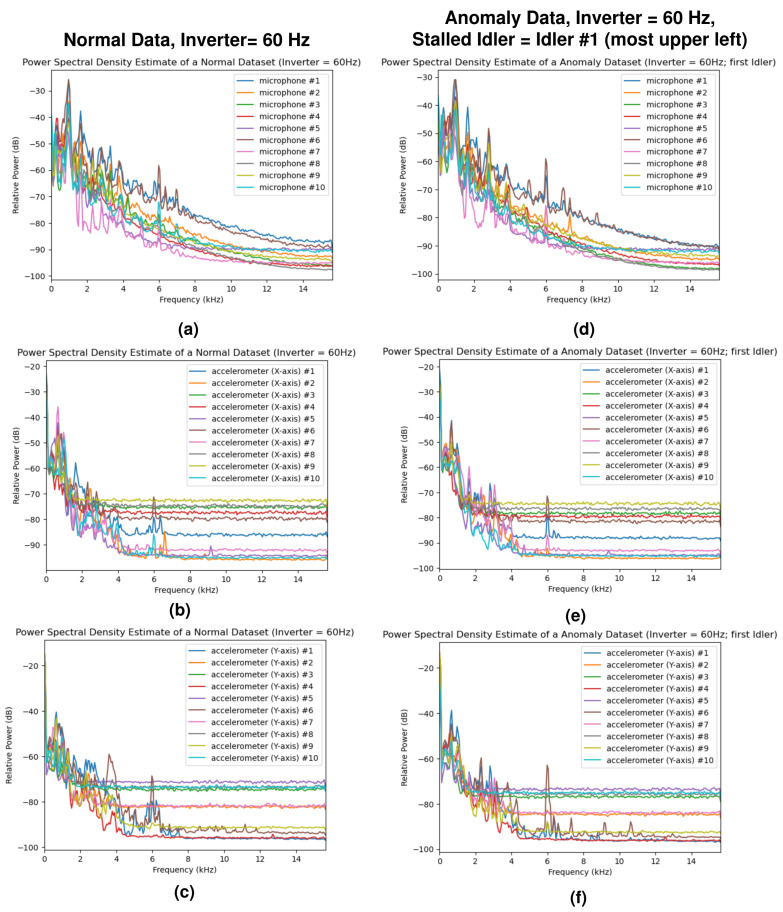
Power spectral density (PSD) estimates of the sensor data for an inverter frequency of 60 Hz: (**a**,**d**) PSD estimates of the microphone sensors for the normal and anomaly datasets, (**b**,**e**) PSD estimates of the *x*-axis accelerometer sensors, and (**c**,**f**) PSD estimates of the *y*-axis accelerometer sensors. The sensor numbers (#1–#10) in the plots above correspond to the sensor identification numbers in Figure 3.

**Figure 7:**
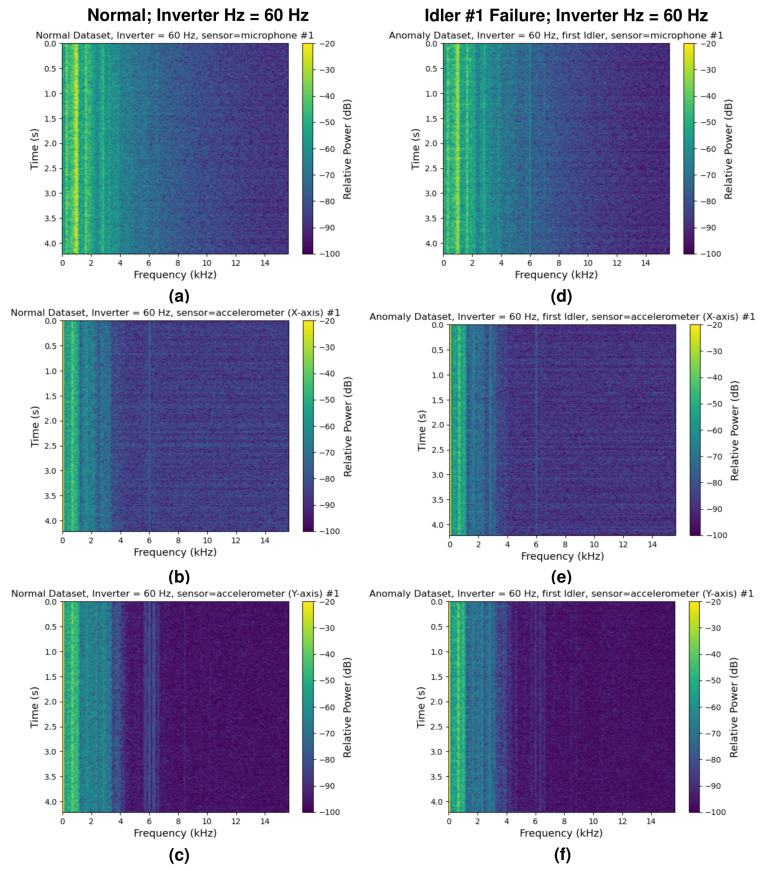
Spectrograms of the first sensor unit in the case of the normal operation (**a**–**c**) and in case where the nearest idler is stalled (**d**–**f**) for an inverter frequency of 60 Hz; (**a**,**d**) spectrograms of the microphone, (**b**,**e**) spectrograms of the *x*-axis accelerometer sensor, and (**c**,**f**) spectrograms of the *y*-axis accelerometer sensor. The sensor numbers (#1–#10) in the plots above correspond to the sensor identification numbers in Figure 3.

The power spectral density (PSD) estimates of the normal dataset and anomaly dataset (idler #1 stall, which is the nearest idler to the sensor unit #1) at the inverter frequencies of 10 Hz and 60 Hz are shown in Figure 5 and Figure 6, providing a visualization of the collected data. In addition, the spectrograms of the first sensor unit during normal operation and during the idler #1 stall are shown in Figure 7. While there are some minor differences in the plots, distinguishing the normal data from the anomaly data is difficult when using only the PSD estimates. The STFT spectrograms are easier to distinguish, as the spectrograms from the anomalous samples contain intermittent broadband frequency components.

### 3.3. Model Performance

The classification accuracy of the model is shown in Table 3 and Table 4, along with the effect of data augmentation (channel masking). The classification accuracy of the model is reasonably high even when exposed to an unseen domain (inverter frequency of 30 Hz).

#### 3.3.1. Effects of Data Augmentation and Sensor Failures

The classification accuracy results of the models trained with and without channel mask augmentation are displayed and compared in Table 3 and Table 4. Three different configurations were evaluated: with every sensor channel available, with 20% of the sensor channels masked by zeros, and with 80% of the sensor channels masked by zeros.

The results displayed in Table 3 were calculated using test samples from the observed environments (inverter frequencies of 10–20 Hz and 40–60 Hz), while the results in Table 4 were calculated with samples from the unseen environment (inverter frequency of 30 Hz), which were excluded from the training dataset. The results on the unseen environment show that resiliency of the model trained with augmentation is vastly better than the model trained without augmentation.

#### 3.3.2. Effects of the Input Data Dimensions

Next, we evaluated the effects of the input data dimensions on the accuracy of the model. The choice of input dimensions can affect the accuracy of the model along with the network bandwidth, energy requirements, and/or classification intervals.

The results in the Table 5 were obtained by progressively reducing the number of the time series accelerometer and microphone samples used to detect and identify faults. The DFT size and the overlap length of the STFT operations were left unmodified. It can be seen that the accuracy starts to drop quickly when the input dimensions are reduced below 30 × 64 × 256.

#### 3.3.3. Effects of Decimation and Downsampling

This subsection evaluates the effects of downsampling the data samples. This knowledge can allow us to better understand the sampling rates and analog bandwidth requirements of the sensor units, which can be used to optimize the network bandwidth requirements of the system and permit the use of inexpensive lower-bandwidth sensor hardware.

Two different downsampling configurations were evaluated, namely, downsampling with and without a low-pass filter. In the former case, an ideal integer downsampler with an ideal (square) low-pass filter was simulated by calculating spectrograms and dropping the high-frequency components from the calculated spectrograms. The resulting spectrograms were then directly input to the CNN models for identification. In the latter case, the raw samples were directly decimated without applying a filter. This causes high-frequency components in the samples to be down-aliased. While aliasing is often considered to be bad, it can actually help the system to retain its classification accuracy when the spectrum is sparse by behaving as a primitive compressive sensing system.

In the evaluation results shown in Table 6, Table 7, Table 8 and Table 9, the first two tables display the performance of the systems when assuming that every sensor unit is available, while the later two show the performance when assuming that only 20% of the sensor units are available. The models were retrained every time the downsampling configurations were changed, as the downsampling process can remove or shift (by aliasing) the frequency components that are utilized by the model for the classifications.

There are some fluctuations in the evaluation results; the changes in the accuracy are not monotonic when the downsampling factors are increased/decreased, which would have been the case if the system and the models were ideal. This is probably because the CNN models were retrained each time configuration changes were made. The models may have ended up in different local minima due to the randomness involved in the training process. Nonetheless, the overall trends of the results agree with our expectations. The system can definitely cope with somewhat reduced sampling rates, though it is difficult to draw a clear line due to the aforementioned fluctuations.

## 4. Discussion

As shown in Table 3, the accuracy of the implemented model is very high; however, it is not yet ready for real-world deployment. As discussed previously, the scalability of the system still needs to be addressed. In this section, we discuss possible change to the model based on the experimental results presented in the previous section.

### 4.1. Comparison with Previous Works

It is difficult to compare our proposed model against previous works in an objective manner due to the differences in testbed configurations and sensor configurations. In particular, most previous systems have been designed and tested using only a single sensor unit, i.e., a univariate sensing system, which is not suitable for the sensor configurations used in our testbed.

Nonetheless, comparing these prior works with our system can provide invaluable insights about the proposed system. Therefore, we describe the results from previous studies in Table 10 below. Please note that the performance figures in the table below were obtained from the original papers and were not recalculated or re-evaluated with our own datasets, and as such cannot be compared directly.

We have used a relatively simple feature extractor and classifier model compared to other researchers; on the other hand, our proposed system probably has an edge in terms of the quality of the data samples. We have mainly concentrated on the scalability and data quality of the proposed system, while previous researchers placed more effort on optimizing the classification models.

### 4.2. Scalability: Operating with a Subset of Sensors

In Table 3 and Table 4, we show that the resiliency of the model against missing sensor streams can be vastly improved by simply augmenting the training data. While the main rationale behind this was to harden the model against sensor failures, it can be also used to save network bandwidth and energy consumption by allowing the model to operate with only a subset of the sensor units.

By only using a subset of sensor units for inference, the proposed system can save a significant amount of energy and network bandwidth. The results in Table 3 suggest that only four or five out of the ten sensor units are actually needed to make an accurate classification in the case of our configuration. This can be implemented by simply turning off the sensor units and replacing the missing data samples with zeros.

Further generalizations of the model in this aspect could make for an interesting future research topic; for example, having a model that can dynamically select sensor units for inference could make the maintenance and sectorization processes much more flexible. We speculate that it would be possible to implement a reinforcement learning-based sensor controller logic which dynamically connects/disconnects the sensor units as needed to minimize the accuracy loss while maximizing the maintenance interval by conserving the battery to maximize a target utility function. A similar concept has already been proposed and demonstrated, with the authors demonstrating that reinforcement learning can be used to select the best subset of temperature sensor units for monitoring the temperature of a given area [27].

### 4.3. Scalability: Bandwidth and Energy Considerations

As shown in the previous section, the amount of data needing to be transmitted by our idler stall identification system is 7.5 MiB per inference (0.75 MiB per sensor unit) when no data reduction techniques are applied. Assuming a Wi-Fi Halow-based network backhaul, this means that approximately 0.76 Wh of energy is used per transmission (0.076 Wh per sensor unit).

The energy costs of the other parts of the sensor hardware are usually much lower, and consequently mostly negligible: the ADXL1002 accelerometer needs approximately 0.005 W [18], the microphone module uses approximately 0.05 W [19,28], and the ADC and MCU boards use approximately 0.038 W and 0.1 W of power, respectively (assuming an ADS1256 ADC and STM32F0512 MCU) [29,30]. Therefore, the combined power draw of the sensor unit, excluding the wireless components, is approximately 200 mW (pessimistically). This means that the sensor unit uses 0.2 Wh of energy per hour, assuming that every chip is always in full operation. Meanwhile, the energy required for a sample transmissions is approximately 0.076 Wh per inference, which can occur up to tens of times per minute if no duty cycling is used.

If a sensor unit has a 3000 mAh 3.7 V Li-ion battery installed, a brand-new battery would last for

(7) $$\frac{3\;\mathrm{Ah}\times 3.7\;V}{0.076\;\mathrm{Wh}}=145\;\mathrm{inferences}$$

counting only the energy used for wireless data transfer. This means that without mainline power or a large solar panel, the battery of the sensor unit may quickly drain if no data reduction is applied.

The simplest method to reduce this energy cost is to duty-cycle the sensors, that is, to slow down the inference intervals and put the sensor units into sleep mode whenever inference is not in progress. While this is one of the most commonly used power-saving methods [11], it comes at the cost of slower identification speed and slightly reduced sensitivity of the system due to the reduced number of inferences made in a unit of time. Thus, while duty cycling can increase the battery lifespan and scalability of the system to almost an arbitrary scale, its use must be minimized whenever possible.

Fortunately, there are many other opportunities to reduce the bandwidth requirements of the system. For example, the results in Section 3.3.2 and Section 3.3.3 (Table 6, Table 7, Table 8 and Table 9) suggest that the sampling rates and length of the input data of our system are somewhat redundant. Because the network bandwidth and energy requirements are directly proportional to these parameters, simply cutting the sampling rates to a quarter and the sample lengths to half would reduce the associated costs by one-eighth. Even further reductions would be possible with some loss of accuracy. When paired with a high-throughput network backhaul (such as 5G NR), the one-eighth reduction rate would allow the system to support approximately 1000–2000 sensor channels concurrently.

By combining duty cycling with downsampling and downsizing techniques, the network efficiency and the battery life of the sensor units can be significantly boosted. For example, if the sensor unit were duty cycled in a such way that it would transmit one data sample per hour and the data rate reduction of one-eighth were applied to the sensor, the sensor would last approximately

(8) $$\frac{3\;\mathrm{Ah}\times 3.7\;V}{(0.076/8)\;\mathrm{Wh}}=1168\;\mathrm{inferences},$$

which is approximately 1168 h, or 48 days. With larger battery modules and/or further data reductions (1/16 reduction with subset selection, for example), it would be possible to improve the battery life to approximately a year.

One of the insights obtained from the spectrograms of our sensor data (Figure 7) is that the anomalous data samples often contained intermittent wideband frequency components (Figure 7d) which were not present in the normal samples. This could explain why the STFT-based model showed a higher accuracy over the alternative designs that we considered during our preliminary analysis. STFT can capture both the short-term power estimates and the long-term power fluctuations, which were not captured well by other preprocessing techniques we tested, such as continuous wavelet transforms. If this is indeed the case, then designing simpler handcrafted features that capture both aspects of the spectrum could allow for further reduction of the data rates. Cyclostationary analysis techniques such as those in [31] could also prove helpful if any repetitive patterns could be found in the measurements.

### 4.4. Long-Term Stability

One of the challenges that is unaddressed in this article and left as future work is improving the long-term stability of the system. A common problem of supervised fault detection models such as the one proposed in this article is that the accuracy of the system tends to degrade over time. This is because such models are often not designed with environmental variables in mind; as time passes, the surrounding environment often changes, and the various properties of the system, including the conveyor and the sensors themselves, may undergo drift as they age. While this would not happen in an ideal world where the model is trained with an infinite amount of data samples from various domains, real-world datasets are much more restricted and biased, causing the model to overfit to specific conditions.

One way to overcome this problem is to use handcrafted features designed to be mostly independent from external environmental factors and aging of the hardware. An alternative approach to solve this problem would be to use unsupervised or semi-supervised learning techniques, which would allow the model to be automatically retrained using more recent unlabeled samples as needed and without human intervention. One such example is an autoencoder-based unsupervised anomaly detection model consisting of an autoencoder model (a deep learning-based lossy data compression–decompression model) intentionally overfitted to normal operating conditions. Because the model is overfitted to normal conditions, it does an excellent job of compressing normal samples, but does not compress abnormal samples as efficiently. This can be used to detect the presence of anomalies [32]. The training process is mostly unsupervised, as the system (the conveyor) mostly operates in normal condition; thus, most of the collected data samples are from these normal operating conditions, and the model remains likely to overfit to them even when abnormal samples are not filtered out carefully.

Continual learning could also be a possible solution to the same problem. As the name suggests, continual learning is a technique involving continuous training and updating of the model even after it is deployed in the field. In this case, the original training datasets are usually not expected to be available. Common workarounds to the problem include generating substitute samples using generative networks or carefully configuring the regularization factors to prevent the model from experiencing catastrophic forgetting. Recently, there have been attempts to bring such techniques to the world of anomaly detection systems, for example, in [33]. Implementing such systems can help the model to retain its performance even over a long period.

## 5. Conclusions

Conveyor belt idlers are cylindrical supports used to support the belts of belt conveyor systems, and can cause major damage to the belts as they fail. Therefore, early detection of idler failures is crucial to maintaining the condition of belt conveyor systems. In this article, we have implemented and evaluated an idler stall detection system using our belt conveyor testbed equipped with accelerometer and microphone sensor units. The scalability of the implemented system in terms of resilience to sensor failures, network bandwidth requirements, and energy requirements is carefully analyzed. The results suggest that our proposed system can easily cope with a few sensor failures, and can be scaled up to meet the requirements of larger belt conveyor installations; however, the energy budgets of the sensor units must be carefully considered, as wireless data transmissions can quickly drain the batteries of the sensor units.

Future research topics involving the proposed system include the possibility of dynamically connecting/disconnecting the sensor units to the network to conserve battery life, as well as the possibility of long-term performance variations of the model and workarounds/fixes, if needed.

## Figures and Tables

**Table 1:** Collected datasets for the belt idler stall detection experiments.

Condition	Inverter Frequency (Hz)						Sum
	10	20	30	40	50	60	
Normal	11 h	11 h	11 h	11 h	11 h	11 h	66 h
Stalled Idler	20 min × 14 idlers	20 min × 14 idlers	20 min × 14 idlers	20 min × 14 idlers	20 min × 14 idlers	20 min × 14 idlers	28 h

**Table 2:** Default hyperparameters and configurations used to train the proposed idler stall identification model.

Name	Value
Input Shape	30 × 256 × 256
Preprocessing Methods	STFT
Augmentation	channel mask
Batch Size	16
Learning Rate	0.0005
Epochs	50 (with early stopping)
Dataset Split	8:1:1 (train:val:test)
Loss Function	Cross Entropy loss
Optimizer	Adam

**Table 3:** Model accuracy evaluation results with samples from observed environments (inverter frequencies of 10–20 Hz and 40–60 Hz).

Channel Mask (% Masked)	Model Training Configurations	
	Without Augmentation	With Augmentation
No Mask	99.522%	99.952%
10% masked	7.321%	99.856%
20% masked	6.770%	99.450%
30% masked	7.201%	99.474%
40% masked	4.833%	99.593%
50% masked	3.469%	99.761%
60% masked	3.469%	98.876%
70% masked	3.469%	97.584%
80% masked	3.469%	91.340%
90% masked	3.469%	58.158%

**Table 4:** Model accuracy evaluation results with samples from the unseen environment (inverter frequency of 30 Hz).

Channel Mask (% Masked)	Model Training Configurations	
	Without Augmentation	With Augmentation
No Mask	96.339%	97.100%
10% masked	7.237%	99.783%
20% masked	10.330%	96.653%
30% masked	4.869%	95.360%
40% masked	3.467%	88.740%
50% masked	3.443%	88.232%
60% masked	3.443%	83.279%
70% masked	3.443%	83.569%
80% masked	3.443%	75.933%
90% masked	3.443%	49.305%

**Table 5:** Model accuracy evaluation results with varying input data dimensions.

Input Dimensions (ch × len × DFT_dim)	Accuracy (Seen)	Accuracy (Unseen; 30 Hz)	Transmitted Data (Per-Sensor)	Transmission Energy (Calculated, Per-Sensor)
30 × 512 × 256	100.0%	96.537%	1.5 MiB	0.152 Wh
30 × 256 × 256	100.0%	96.230%	0.75 MiB	0.0762 Wh
30 × 128 × 256	99.95%	94.177%	0.375 MiB	0.0381 Wh
30 × 64 × 256	99.36%	83.467%	0.188 MiB	0.0190 Wh
30 × 32 × 256	88.665%	68.961%	0.094 MiB	0.0095 Wh

**Table 6:** Model accuracy evaluation results with downsampled accelerometer/microphone samples (no low-pass filter applied).

Downsample Factor	Input Dimensions (ch × len × DFT)	Accuracy (Seen)	Accuracy (Unseen; 30 Hz)	Transmitted Data (Per-Sensor)
×1	30 × 256 × 256	99.904%	97.100%	0.75 MiB
×2	30 × 256 × 128	99.833%	91.929%	0.375 MiB
×4	30 × 256 × 64	99.904%	82.711%	0.188 MiB
×8	30 × 256 × 32	99.856%	91.253%	0.094 MiB
×16	30 × 256 × 16	96.938%	91.966%	0.047 MiB
×32	30 × 256 × 8	96.675%	76.392%	0.023 MiB
×32	30 × 32 × 64	99.737%	79.352%	0.023 MiB

**Table 7:** Model accuracy evaluation results with downsampled accelerometer/microphone samples when applying an ideal DFT low-pass filter.

Downsample Factor	Input Dimensions (ch × len × DFT_dim)	Accuracy (Seen)	Accuracy (Unseen; 30 Hz)
×1	30 × 256 × 256	99.952%	97.100%
×2	30 × 256 × 128	99.904%	91.699%
×4	30 × 256 × 64	99.330%	88.208%
×8	30 × 256 × 32	97.464%	97.378%
×16	30 × 256 × 16	98.780%	81.624%
×32	30 × 256 × 8	94.952%	83.835%
×32	30 × 32 × 64	97.967%	76.754%

**Table 8:** Model accuracy evaluation results using downsampled accelerometer/microphone samples (no low-pass filter applied) with eight out of ten sensor units disabled (masked).

Downsample Factor	Input Dimensions (ch × len × DFT_dim)	Accuracy (Seen)	Accuracy (Unseen; 30 Hz)
×1	30 × 256 × 256	77.751%	67.645%
×2	30 × 256 × 128	81.746%	64.589%
×4	30 × 256 × 64	81.220%	74.931%
×8	30 × 256 × 32	70.454%	52.906%
×16	30 × 256 × 16	71.770%	80.766%
×32	30 × 256 × 8	62.249%	66.689%
×32	30 × 32 × 64	65.646%	51.371%

**Table 9:** Model accuracy evaluation results, using downsampled accelerometer/microphone samples (ideal DFT low-pass filter applied) with eight of ten sensor units disabled (masked).

Downsample Factor	Input Dimensions (ch × len × DFT_dim)	Accuracy (Seen)	Accuracy (Unseen; 30 Hz)
×1	30 × 256 × 256	77.727%	67.657%
×2	30 × 256 × 128	95.789%	63.115%
×4	30 × 256 × 64	73.493%	62.305%
×8	30 × 256 × 32	67.201%	66.075%
×16	30 × 256 × 16	69.019%	62.921%
×32	30 × 256 × 8	67.727%	54.633%
×32	30 × 32 × 64	80.167%	49.583%

**Table 10:** Comparison of the proposed model against previous works. Note that the accuracy measurements have not been recalculated using our datasets.

Parameter	Ours	Alharbi, F. [5]	Yang, M. [6]
Year	2024	2024	2020
Sensor Type	Accel + Microphones (multivariate)	Microphones	Microphones
Feature Extraction	STFT	BPF, Neural Networks (YAMNet, BiLSTM)	varies (handcrafted, autoencoder, etc.)
Classifier	CNN (MobileNet V2)	XGBoost	varies (SVM, DNN, CNN attempted)
Sample length	4.2 s	0.48 s	14 s
Accuracy ^1^	99.9% (best case)	approx. 95% ^2^	96.7%

^1^ Results as reported in the original paper. ^2^ The goal of the classifier is slightly different from ours (failure stage estimation).

## Data Availability

The datasets presented in this article are not readily available due to an another ongoing study with overlaps in the dataset. Requests to access the datasets should be directed to the authors of the article.
